# Designing a conversational agent to promote teamwork and collaborative practices using design thinking: An explorative study on user experiences

**DOI:** 10.3389/fpsyg.2022.903715

**Published:** 2022-10-11

**Authors:** Sofie Smedegaard Skov, Josefine Ranfelt Andersen, Sigurd Lauridsen, Mads Bab, Marianne Bundsbæk, Maj Britt Dahl Nielsen

**Affiliations:** ^1^National Institute of Public Health, University of Southern Denmark, Copenhagen, Denmark; ^2^Gnist, Aarhus, Denmark

**Keywords:** mental health, employee wellbeing, occupational psychology, teamwork, design thinking, participatory intervention

## Abstract

Appearance, voice features, and communication style affect users trust in conversational agents (chatbots), but few studies have assessed what features users like and dislike. Using design thinking, we developed Susa, a conversational agent, to help workplaces promote teamwork and collaborative practices. Design thinking prioritizes co-creation and multidisciplinary teamwork to develop innovative solutions to complex problems. The aim of this qualitative study was to explore users’ interactions with and reactions toward Susa and explain how we used user inputs to adapt and refine the first prototype. The employees and managers from four workplaces participated in three workshops to test and refine the agent. We applied an explorative thematic analysis of data collected *via* video recordings of the workshops. The results of the analyses revealed that visual identity, communication style and personality was important for acceptability. Users favored a more human like agent that primarily communicated with the team *via* text messages. Users disliked emoticons and humor because these features clashed with the seriousness of the topic. Finally, users highlighted that Susa helped structure organizational change processes, develop concrete action plans, and stay on track. It is a weakness that Susa is a simple robot based on a preprogrammed script that does not allow users to adapt the process.

## Introduction

Conversational agents, also known as virtual agents and chatbots, are computer programs designed to simulate human text or verbal conversations. Research shows that users can relate to conversational agents in a social way, as if the agents were human (Gaffney et al., 2019; Vaidyam et al., 2019). Appearance, voice features and communication style affect users trust in the agent, and trust plays a key role in adoption and long-term utilization (Loveys et al., 2020). However, previous studies have produced somewhat conflicting results about what features users like and dislike (Milne-Ives et al., 2020), and users may perceive design feature differently depending on the setting and the tasks performed by the agent (Loveys et al., 2020). To improve the usability and acceptability of conversational agents, tailoring the design specifically to the intended target population is key (Nadarzynski et al., 2019).

In this study, we develop a simple conversational agent named Susa to help small- and medium-sized enterprises (SMEs) strengthen their teamwork and promote collaborative practices characterized by shared goals, mutual respect, timely communication, and knowledge sharing (Bolton et al., 2021). There is no unifying theory explaining the complex relationship between the psychosocial working environment and mental health and wellbeing at work. More recent theories, e.g., on organizational justice, social capital, and relational coordination, focus explicitly on social relations, and empirical research has shown that positive relations at work are related to better mental health, while low social support, a lack of trust, and conflicts increase the risk of mental health problems and sickness absence. At the same time, social support and collaborative practices have also been related to higher productivity, work engagement and job satisfaction (Nieuwenhuijsen et al., 2010; Schutte et al., 2014; Nielsen et al., 2016; Rugulies et al., 2020). Thus, over the past decades much scholarly attention has been directed toward facilitating management practices and cooperative patterns that improve production process and the wellbeing of employees simultaneously (Meng et al., 2019).

We developed Susa for and together with small- and medium-sized enterprises (SMEs) inspired by principles of design thinking. Design thinking prioritizes deep empathy for end-user’s needs to fully understand a problem and to develop cross-disciplinary solutions to complex problems (Roberts et al., 2016; Micheli et al., 2018). Design thinking has attracted considerable interest from practitioners and academics alike, as it offers a novel approach to innovation and problem-solving, especially when addressing wicked problems (in the sense of being ill-defined or tricky). Although different terms and sequences of action are employed in design thinking models, e.g., in applied models such as IDEO, Stanford Design School and IBM, a certain degree of commonality exists (Micheli et al., 2018). These models tend to start from an initial exploration with the objective of understanding the problem to be solved. They then move onto an ideation stage to generate alternatives, and conclude with an implementation and testing phase, based on prototyping and iteration (Micheli et al., 2018).

We chose to focus specifically on SMEs, because SMEs pose a challenge for occupational health and safety. SMEs are less likely to comply with occupational health and safety regulation, i.e., to assess risk factors at work and initiate preventive efforts, and few occupational health and safety interventions have been designed with a proper understanding of the challenges facing SMEs (Legg et al., 2015). First, SMEs have more limited resources to prioritize occupational health and safety compared to large enterprises (Legg et al., 2015). This means that they may not be able to hire external consultants to help facilitate interventions. Second, they rarely posit the necessary expertise in house, e.g., occupational health and safety experts and HR personnel. Third, SMEs are a particularly hard to reach group—even in well-designed interventions. The main reasons are the high cost of delivering interventions through personal contact, which is preferred by SMEs.

Interventions that seek to improve the psychosocial working environment (i.e., related to interpersonal relations, such as teamwork and collaborative practices) are often complex, and implementation is challenging, even in larger enterprises. Successful implementation typically relies on a high level of guidance and facilitation from researchers, consultants, and occupational health and safety experts. These consultants and experts play an important role, e.g., by assuming the responsibility to convene meetings, acting as a third party and facilitator between employers and employees, lending credibility to the intervention and bringing out new perspectives on problems and solutions (Jauvin and Vézina, 2015). However, the dependence on external experts threatens long-term sustainability, as interventions often collapse, when experts leave (Nielsen et al., 2010; Jauvin and Vézina, 2015; Meng et al., 2019). Thus, to enable and empower managers and employees to improve teamwork and collaborative practices, we designed Susa to imitate an external facilitator to make workplaces less dependent on human facilitation.

In brief, Susa guides a team of employees and managers through a process of identifying a challenge related to teamwork and collaborative practices and developing and implementing solutions (we describe the process and functionalities in-depth in section Susa: Background and functionality). This bottom-up approach is inspired by participatory workplace practices that allows employers and managers to come together to develop their own solutions. Previous research consistently shows that a high level of participation and support from both managers and employees is key for successful adoption and implementation of occupational health and safety interventions. Moreover, making use of employees’ job expertise and knowledge of the organizational context also provides an important supplement to the expertise of intervention experts and can help tailor interventions to the specific culture and needs of the workplace. Because participatory methods treat employees as co-learners, they may also add an element of respect, esteem, and reward for participants (Nielsen et al., 2010; Abildgaard et al., 2020).

In this paper, we explore users’ interactions with and reactions toward Susa and explain how we used user inputs during the design process. We focus specifically on users’ reactions to Susa voice and communication style, visual design and personality and relationship with Susa.

## Susa: Background and functionality

**Figure 1:**
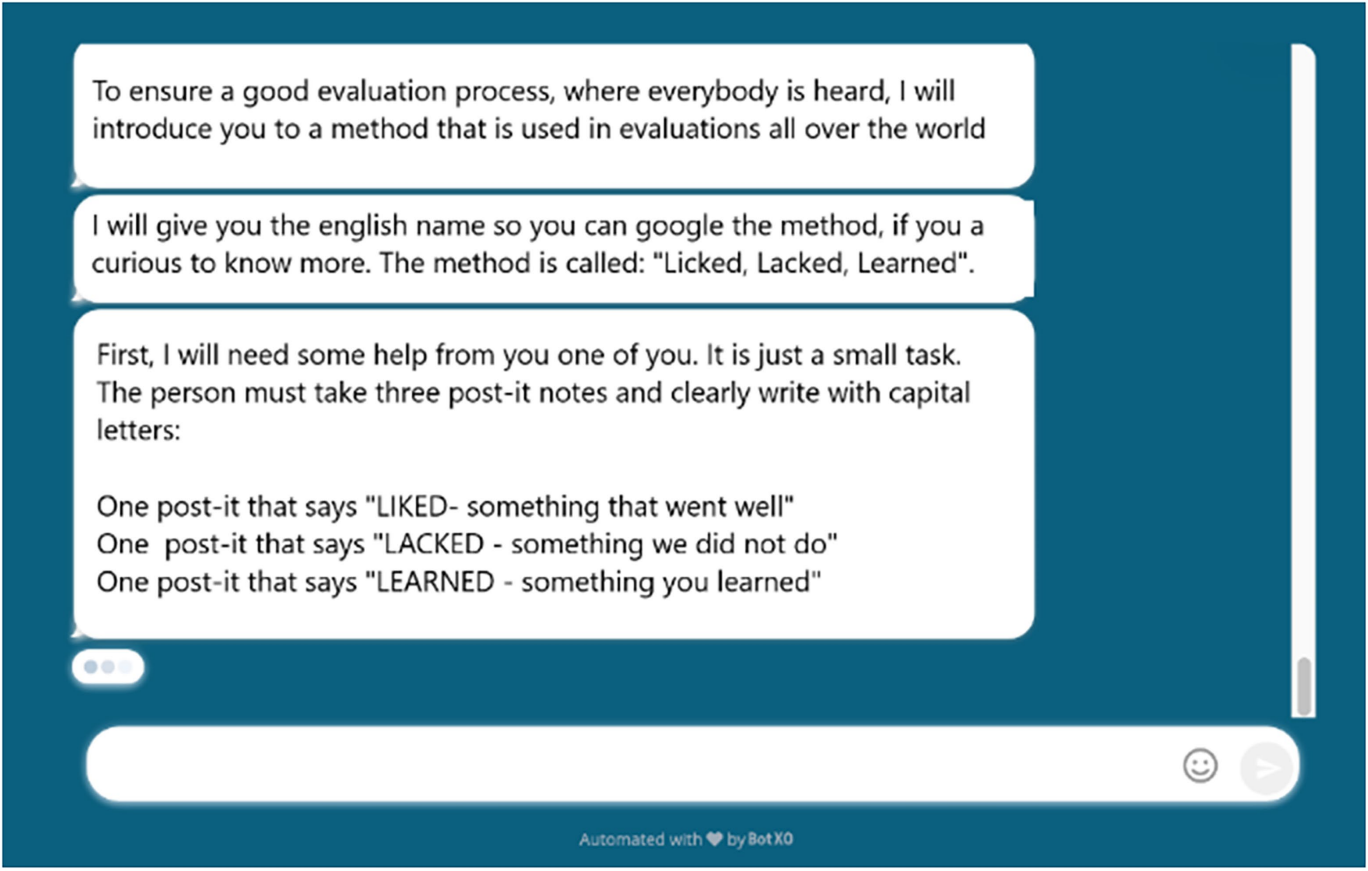
Chat window.

Based on previous research by Nielsen et al. (2010), we identified common steps that are central for participatory interventions: (1) identification of workplace challenges and problems; (2) identification of solutions to problems; (3) development of action plans that specify who does what when; and (4) implementation and evaluation. Using these steps as a framework, we designed Susa to guide a team of employees (and preferably one or more managers) through a process consisting of three team meetings (lasting about 1 h each). The team decides when and where these meetings will take place allowing a high degree of flexibility in the process. The meetings, however, require that team members be together physically and have a computer, tablet og smartphone at their disposal. The team must assign one coordinator, who are responsible for communicating with Susa during the meetings. The coordinator (and the team) communicates with Susa using preprogramed options in a specially designed chat window (shown in Figure 1). The chat window makes it possible to display images, visual instructions, and animated timers alongside Susa’s text instructions.

Susa explains the process and introduces the different activities and assignments at each meeting (either *via* small videos or text messages in the chat window). Susa also helps facilitate the meeting, e.g., keeps track on time and ensures that everyone is allotted the same amount of time to speak. During the first meeting, the team must select a challenge or a problem they want to work with. The team can address any topic or challenges they wish but are informed that Susa’s specialized in teamwork and collaboration in the first introductory video were Susa also presents herself and the process to the team. During the second meeting the team identifies relevant solutions and lay out an action plan; and follows up and evaluates the implementation during the third meeting (following the framework outlined above). Table 1 shows the main activities during each meeting.

**Table 1:** Overview of team activities during team meetings.

**Meeting 1: Identification of challenges**
Susa explains the process and advices the team to stay on time, to stay focused, and think about when to speak (and not). The team can choose to proceed to their first task or to see a couple of examples of challenges for inspiration. Team members brainstorm individually and writes down challenges on a post-it, and present two of their challenges to the others. Next, the team sorts the challenges, removes duplicates, and votes and selects two challenges they want to work with. The team brainstorms individually on possible causes and present their analysis to the teak. The team decides if they need to change their challenges and evaluates the process. Finally, the team record their challenge(s) in the chat window, and Susa gives a brief introduction to the next meeting.
**Meeting 2: Identification of solution and development of action plans**
Susa introduces the tasks ahead, and the teams challenge(s) appears on the screen (based on their input from last meeting). Susa asks the team to briefly discuss why this challenge was important. The team members write down solutions on post-its (as many as possible), and each person selects one solution that they present to each other. The team must choose one solution (each team member gets three votes). Susa explains why it is important to be very concrete, and team members engage in a silent brainstorm to think about concrete steps and actions. They must note down 1–5 actions that they believe can be implemented within 2–4 weeks. The team presents their post-it is and selects 1–5 actions. The team writes their actions in the chat window and indicate who will implement and when.
**Meeting 3: Evaluation**
Susa introduces the tasks ahead, and the teams input from the last meeting appear on the screen. Each person must evaluate themselves on a scale from 1 to 10 (to what extent they have implemented their tasks) on a post-it. The team calculates the average assessment and enter their score in the chat window. Susa gives the team feedback based on their score. Next, each team members write downs things that went well (liked), things they did not do (lacked), and things they have learned (learned) on post-its. Next, the team organize their post-it is in three groups (liked, lacked, learned), and discuss them. Finally, the team decides what to do next.

## Materials and methods

Inspired the guidelines for reporting health research involving design (Bazzano et al., 2020), we describe the composition of the project team, the involvement of users, and outline the main activities and procedures for data collection and data synthesis. Table 2 gives an overview of the project phases and activities. This paper does not include phase 4, which will include an effect evaluation to assess changes in short term outcomes related to collaborative practices (e.g., changes in shared goals, role clarity, respect, and social support) using the Danish Psychosocial Work Environment Questionnaire (Clausen et al., 2019), and more long-term outcomes related to mental health using the short version of the Warwick-Edinburgh Mental Wellbeing Scale, also known as SWEMWS. SWEMWS was developed to monitor and evaluate programs to improve mental wellbeing in the general population. The scale covers both feeling and functioning aspects of mental wellbeing including hedonic (positive feelings, affect, emotions) and eudemonic (positive functioning, mindset and relationships) aspects (Koushede et al., 2019).

**Table 2:** Overview of the design process.

Phase (year)	Phase 1 Understand (2019)	Phase 2 Explore (2019)	Phase 3 Prototype (20–2021)	Phase 4 Evaluate (2022–2023)
Participants	Researchers, (developers), and users^1^	Developers (and researchers)	Developers, graphic designers, software engineers, (researchers), and users^1^	Researchers and users^1^
Activities	Workshops and interviews	Brainstorming sessions and meetings	Tests of prototypes and feedback from users (workplace labs)	Data collection and analyses based on surveys and interviews
Data collection	Audio recordings	Pictures	Video recordings	Survey among 30 SMEs and interviews among 10 SMEs
Synthesis and analysis	Summaries to project team	Minutes of meeting	Summaries to project team Paper	Evaluation reports Paper

1 Employers and employees from SMEs.

### Participants and partners

#### The project team

The project team consisted of researchers specialized in mental health and working environment research from the National Institute of Public Health (NIPH) in Copenhagen Denmark and consultants from Gnist in Aarhus specialized in organizational development. In the remainder of this article, we refer to the two partners as the researchers (NIPH) and developers (Gnist). As shown in Table 1, the researchers were primarily responsible for the activities in the understand and evaluation phase, whereas the developers were responsible for the activities in the explore and prototype phase. However, both partners contributed to all phases and drafted the initial project description together. Later, the team solicited a professional designer and a company specialized in conversational agent to help with the development of the prototype of Susa.

#### Participant’s characteristics

We invited 10 SMEs to participate in the project. The participants were recruited *via* the team member’s own network. We only included SMEs from the private sector with less than 150 employees, as this was a funding requirement, and purposefully selected SMEs from different industries to achieve maximum variation. The characteristics of the ten SMEs, which all participated in phase 1, are shown in Table 3. We invited five SMEs to participate in phase 3, four of these accepted (one SMEs dropped out during phase 3). We purposefully selected the SMEs based on the data collected during the interviews (phase 2). The selection criteria included: motivation and engagement in the study, managers and employee’s belief in the possibility to change things at work, digital maturity, and psychological safety. We established these criteria to increase the chance that the SMEs would be able to actively participate in the design process and reduce the risk of drop-out.

**Table 3:** Participant characteristics.

ID	Size^*^	Industry	Engagement in project
WP1	50–99	Business consulting	Phase 1
WP2	20–49	Call center	Phase 1
WP3	10–19	Pharmacy	Phase 1 and 3
WP4	20–49	Engineering	Phase 1
WP5	10–19	Communication	Phase 1 and 3
WP6	20–49	Production	Phase 1
WP7	10–19	Veterinarian	Phase 1 and 3
WP8	20–49	Property management	Phase 1
WP9	20–49	Accounting	Phase 1
WP10	20–49	Electric Installation	Phase 1 and 3

* Number of employees.

### Using design thinking in this study

In this project we followed the main steps as outlined in the IBM framework, which we will briefly describe below. While this framework includes a series of distinct phases and steps, in practices the process is iterative. The IBM framework outlines four phases: (1) Understand, (2) Explore, (3) Prototype, and (4) Evaluate. In the understand phase, the project team engages with users to better understand their needs and problems, e.g., through observations, interviews, and questionnaires. This phase ends with development of a meaningful and actionable problem statements. The explore phase focus on the generation of innovative ideas to avoid obvious spoliations and to increase the innovation potential, e.g., using brainstorming to leverage the collective thinking of the team, by engaging with each other, listening, and building on other ideas. The protype phase is the iterative generations of artifacts intended to answer questions to solve the design problem, e.g., using mockups that support the elaboration and evaluation of the product. The goal of this phase is to validate the ideas proposed during the explore phase. In the evaluation phase the team solicits feedback from users (Lucena et al., 2017).

#### Understand (phase 1)

The understand phase consists of different sets of activities to help understand users and to gain a deeper understanding of how they think (Lucena et al., 2017). In this project, we retrieved input from previous research and semi-structured interviews with 10 SMEs. We conducted interviews with 39 employees and 18 mid-level and top managers/directors from 11 March to 10 April 2019. Interviews with managers and employees were conducted separately to allow employees to speak more freely. The interviews followed a semi-structured interview guide. In addition, we introduced the interviewees to different themes, e.g., trust, communication, and coordination, written on small pieces of papers, and asked the interviewees to select at least three themes relevant for their workplace (or to write down their own themes). After each interview, the researchers prepared a written summary, which was shared with the rest of the team. All interviews were audio recorded and subsequently transcribed, which will allow us to do an in-depth qualitative analysis at a later point. In this section, we focus on how we used user inputs based on the summaries to guide the design process.

The team discussed the results from the summaries, and especially two issues stood out. First, it was clear that each workplace had their own unique challenges, although we did identify some common themes, including communication, psychological safety, constructive feedback, coordination, and common goals and knowledge sharing. Second, we found that managers and employees from the same workplace often had different understandings of the extent and causes of challenges and problems. These findings underscored a need to develop a flexible solution that could be tailored to the needs of the individual workplaces, rather than developing a solution that could only help with specific challenges, e.g., communication. Moreover, the findings also highlighted a need for bringing managers and employees together to ensure that they have a common understanding of challenges. These insights were also in line with previous literature on participatory workplace interventions as outlined in the introduction.

#### Explore (phase 2)

The purpose of the explore phase is to generate new ideas and possible solutions. To that end, the team engaged in an iterative process consisting of workshops, meetings, and *ad hoc* conversations. Based on input from the previous phase, we decided to build the intervention on participatory principles allowing SMEs to address their unique challenges and problems. The project team also identified the core steps and activities as outlined in section Susa: Background and functionality. Next, the project team discussed different *digital* solutions (defined as an intervention delivered *via* the Internet, e.g., using mobile phones or websites). Although we had decided that the solution should be digital from the onset of the project, we had not specifically decided to use a conversational agent. The developers presented the idea to the project team after meetings with different companies specialized in conversational agents. Finally, after defining the core functionalities of the agent (Susa), the project team developed a program theory that outlined core assumptions about the underlying working mechanisms of the agent.

#### Prototyping (phase 3)

The goal of this phase was to validate ideas proposed during the explore phase. The developers therefore developed the first prototype. The developers tested the opportunity to use artificial intelligence to teach Susa to understand and categorize users’ input, however, this idea was abandoned, because it was not possible to develop a satisfactory solution within this project. This means that we ended up with a rather simple agent with less flexibility to users.

The developers presented the first prototype of Susa to managers and employees from four SMEs at three workshops (hereafter workplace labs) at each workplace. The aim of the workplace labs was to test the prototype and to adjust and refine the agent. The designers continuously adapted and refined Susa but did not change the main structure or functionalities. The changes took place during and after the workplace labs. The first wave of changes focused on improving information about the process and to refine Susa appearance and personality. The second wave took place after finalizing the workplace labs and included simplification of information, and introduction videos for meeting module (some narrated by a human, others by Susa).

##### Workplace labs

Four SMEs participated in the workplace labs. Each workplace appointed a coordinator (typically a manager) and a team of employees. Each workplace labs took place at the workplace and consisted of three workshops designated to test the three modules. During module 1, the team selected one challenge or problem they wanted to improve; during module two the team identified relevant solutions and prepared an action plan; and during module three team followed up and evaluated the implementation of the plan (as explained in-depth in section Susa: Background and functionality). The number of participants at each workshop ranged between 5 and 9 persons. In total, 24 women and 6 men in the age between 25 and 62 participated.

The four SMEs choose to focus on different challenges and therefore also identified different solutions. For instance, one SME wanted to increase role clarity and coordination (WP1), whereas another wanted to enhance employees understanding of the organizational structure and improve collaboration between different departments (WP2), and a third focused on psychological safety and engagement (WP3). Consequently, the different solutions also differed considerably. For instance, WP1 implemented daily morning meetings to make sure that everyone knew what to do, while WP2 implemented new organizational charts. The developer’s primary function was to observe participants engage with Susa and help the team in case of problems.

The researchers were responsible for documenting the process during the workplace labs (described previously). At the end of each module a researcher asked the participants about their experience and their assessment of Susa. Each of the modules were video recorded, while the researcher took observational notes. Later, the data material was processed using the qualitative data analysis software Nvivo. The analysis was inspired by thematic analysis, which is a method for analyzing and reporting patterns (also known as themes) in qualitative data, e.g., interviews and observations (Braun and Clarke, 2006). A theme captures important aspects of the data related to the research question at hand and represents a patterned response or meaning in the data set. The advantage of the thematic analysis is that it can help summarize key features of a large body of data and provide thick descriptions of the data set. It offers a flexible approach to qualitative analysis and is particularly useful for participatory research where participants are key collaborators. The analysis includes a constant iterative process were the researcher moves back and forth between the entire dataset and the coded extracts that are later collapses them into themes. Finally, the researcher reviews the themes, writes up a coherent story, and selects vivid extract examples. Thus, in contrast to statistical analyses, writing is an integral part of the analysis (Braun and Clarke, 2006). In this study, two researchers independently identified and reviewed themes and patterns in the data material and discussed their analyses and observations iteratively. The identified themes all centered around the participants’ reaction and interaction with Susa.

## Results

This section presents the findings from the analysis of participants (user’s) reactions to Susa during the workplace labs. We categorized findings into four themes into the following themes: communication style, visual appearance, personality, and Susa as a facilitator. In the next section, we describe these themes and explain how we used user experiences to adapt and adjust Susa. We refer to two different versions of Susa, because the developers changed Susa according to users input during the workplace labs and after the labs were finalized (as explained previously).

### Communication style

In the first version, Susa primarily relied on audio communication using a very robotic voice. During the first wave of changes, communication shifted to more text-based messages and instructions. Figure 1 shows how Susa instructs the team in an evaluation assignment during the third team meeting. Susa is a simple rule-based agent that depends on prewritten commands programmed by the developer. Consequently, users are therefore restricted to predetermined options when answering questions.

As part of the first wave of changes, the developers gave Susa a more human-like voice, because users disliked the robotic voice and felt that Susa talked too much. For instance, some users felt that it was difficult to listen to the robotic voice, prompting responses such as:” *The voice is too monotone if you have to listen that long” (user).*

Even though users could understand Susa’s instructions, some users experienced difficulties in retaining information, because they found it difficult to concentrate on the robotic voice.

“*I missed a lot because I had to listen to Susa for a long time.[…]. I just don’t get it. I need to use too much energy to understand what she is saying” (user).*

In general, users seemed positive about the changes and felt the more human-like voice was softer and more pleasant, coherent, and trustworthy compared to the old robotic voice: “*It is more pleasant to listen to, and it is real words coming out of her mouth, and the sentences are coherent because she breathes” (user).*

On the other hand, criticism of the new more human voice included that it mumbled too much and talked to fast. Moreover, some users missed Susa speaking, and they seemed less focused on her instructions when there was no sound. The more text-based version of Susa also led some teams to sit silently reading, which may lead some users to become more passive. Several users stated that they would prefer to choose what type of communication to have with Susa, e.g., with or without voice, how much text, the use of emojis etc.

### Visual appearance

Developing a visual identity proved very important for users to relate to the Susa. Most users acknowledged that the visual identity does help to make the process more convincing and familiar, and the visual appearance of Susa sparked a lot of discussions in the teams. As part of the first wave of changes, the developers completely changed Susa’s visual appearance based on users’ reactions. Figure 1 shows the first and second version of Susa. The first version had a more robotic appearance than the second version. In general, users had strong negative reactions toward the first version. For instance, several users found Susa to be too *cute,* that she did not seem serious enough, and that the voice and visual identity did not match:

*“You need to think about how cute she looks. She looks like a cute bubble figure, and that does not really match that we are going to use her for something serious. The professionalism kinds of fades out” (user).*

**Figure 2:**
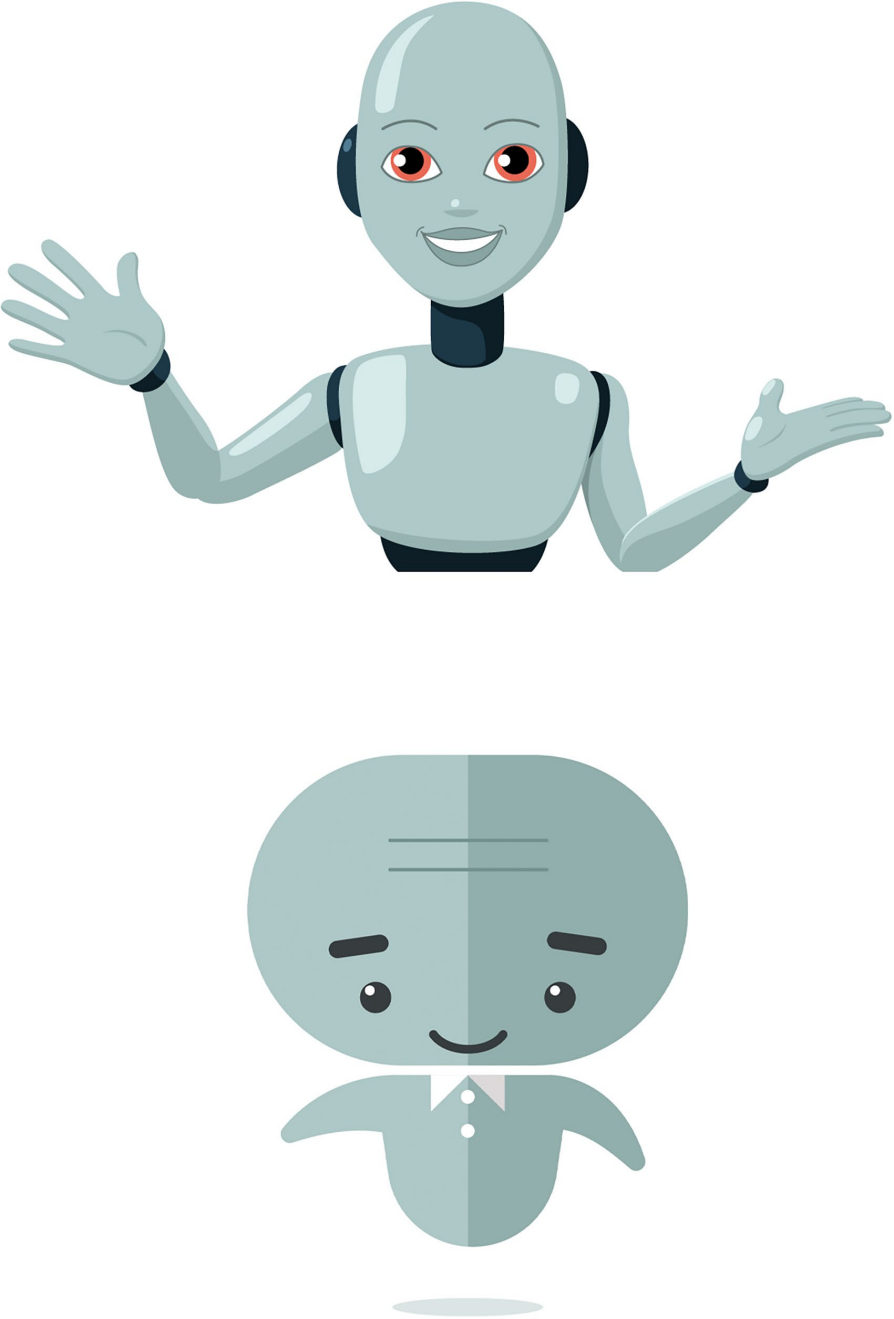
First version (top) and second version (bottom) of Susa.

Although the second version was designed to be more humanlike, some users still felt that Susa was too robotic. For instance, one user said that she seemed like a stereotypical conversational agent and would have preferred much more personality. Some users stated that they would have preferred it if Susa was removed from the chat window after the first meeting, because Susa distracted them. Overall, users reacted strongly to the appearance of Susa in both versions and favored a more human-looking agent (Figure 2).

### Personality

The users also had strong reactions to Susa’s personality. In particularly, users found that the first version of Susa was too cheeky and *too much*, for instance when making jokes and using emoticons, which they perceived as being unnecessary and unserious. Users felt this clashed with the seriousness of the topic and wanted a more professional appearance.

*I don’t need the funny little comments […]. I mean, we are trying to focus on role clarity. This is a professional setting. Here I don’t need those cute cozy little comments” (user).*

Furthermore, users preferred that Susa does not use emojis or encouraging comments but focused on giving instructions instead of wasting time. At the same time, users found personality and *soul* to be important: *“It is important to feel she has some soul, so it is not just another chat platform” (user).*

The video recordings revealed that users seemed to respond more favorably to Susa over time and began to build a relation to her. For instance, some users expressed that they were getting to know knew her better and could therefore more easily follow her instructions. Susa’s personality had a positive influence on a user who exclaimed: “*Aw Susa, you are so nice”* (user).

Some users felt that it was important that Susa was part of the team. They suggested to make a presentation of Susa in the beginning of the first meeting. This introduction could help explain how she can be used and what she can help with. One of the users expressed that the second version of Susa appeared more serious.

### Susa as a facilitator

Reflecting on their experiences in relation with Susa users explained that she was able to facilitate the meetings, and that they felt comfortable following her instructions, although some users found it hard to imagine that a robot could help them with relational challenges in the beginning*: “Our workplace is human, and we deal with emotions, and now we must talk to this—a robot” (user).*

After the revisions explained earlier, users felt that Susa was serious and acted with authority when giving the group assignments and tasks. Moreover, the user trusted Susa’s instructions during the meeting, and found it easy to follow her instruction on the screen.

*I am fan. I think it is fantastic. It is so cool that you get some concrete assignments, it is super nice with the directions” (user).*

As explained earlier, the users seemed able to connect to Susa and one user explained that she would prefer a robot facilitator rather than a human facilitator: “*With Susa the discussion is kept inside the group because you are not talking to an external person you do not know” (user).*

Furthermore, several users liked that Susa summarizes main decisions after each meeting and send out reminders by e-mail to help the team keep on track.

*“I think it is good with the summary…that this is your issue, and this is the desired effects, because with so many things on the table, it is easy to lose our footing” (user).*

While most users felt that Susa contributed positively to the meetings and the process, one experienced manager felt that the help is unnecessary if you are used to facilitate meetings and organizational change processes. Another manager, however, felt that it was easier to stay more present and participate in group discussions, because Susa took over the facilitation of the meetings. Some of the participants noted that it is necessary to have a to co-facilitate from the team to build a bridge between Susa and the team and Susa: “*Susa is a good helping tool–but not yet a moderator” (user).*

The observations also revealed that Susa was not always able to assert the order of dialog between users, which sometimes led some users to overpower the dialog in the group or that a discussion would continue for too long. These scenarios emphasize the need for a co-moderator to help the group get back on track with Susa’s instructions.

While some users felt that the process was rather time-consuming, many acknowledged the importance of not jumping ahead and skipping steps, e.g., immediately jumping to solutions. Thus, Susa helped the teams stay on track and remined them not to move too fast ahead. Moreover, several users appreciated that Susa gave room for individual reflections, and that she gave them time to make up their mind, when making decisions. The democratic process of using voting as a mean of making joint decisions, e.g., about the problem and solution was also highlighted by several users.

*We might not agree if we just sit here and discuss it, it is easier when you vote, because then it is clear what you have chosen” (user).*

Several users highlighted that Susa was fixated on tight time management and asked users to be concrete, when describing challenges and solutions explaining that this is often the reason they have failed earlier, when implementing organizational changes.

“It is just because you have this template, so it does not get so shifty, like when we normally try to solve problems. I often think that you end up with this feeling that yeah right that is not going to happen” (user).

*“We have learned that what makes the process successful is when we can all agree that it is a good solution” (user).*

Being concrete and choosing a realistic solution seemed to be key for the process. While some of the teams in the workplace labs successfully implemented a solution within the project period, others only partially implemented their solution. Susa can only remind users to be concrete, but she cannot evaluate the challenges and solutions and give users feedback on their inputs. Thus, one team realized that they had not been concrete enough at the final meeting, when evaluating the process.

One of other the teams, however, implemented a daily staff meeting in the morning to coordinate and prioritize tasks. Although the need for such a meeting had been on the agenda for years, this was the first time they implemented the meeting in practice. Besides from the benefits of the meeting itself, the team experiencing having joint success, which also boosted wellbeing, and made them feel more confident about implementing changes:

*“When you are successful and cooperate, then it is a positive emotion when you go home happy. I did not even realize that I worked half an hour later yesterday, and…mental health is also about thriving at work” (user).*

## Discussion

Susa represents an innovative approach to occupational health and safety, as the use of conversational agents in this setting is scarce. Wecoach (Grimm et al., 2020; Hungerbuehler et al., 2021) is another example of a virtual agent that aim to increase the capacities of team leaders to promote mental health and wellbeing among team members. Wecoach offers coaching and training sessions to and helps facilitate workshops with employees (Grimm et al., 2020).

Conversational agents have the potential to contribute to health promoting psychosocial working environment, i.e., by reducing risk factors and strengthen positive aspects (Lehr et al., 2016). Although it is unlikely that conversational agents can substitute human occupational experts, conversational agents, like Susa, may automize some services, e.g., convene and facilitate meetings, deliver knowledge, and provide feedback. The potential of such agents, however, will depend on users accepting robots and that they find them helpful and useful. While previous research indicates that conversational agents are acceptable to users, most studies do not report users’ feedback and preferences (Milne-Ives et al., 2020). This study provides new knowledge about the acceptability and useability of conversational agents to promote teamwork and collaborative practices at work. While Susa focuses on interpersonal aspects at work, Susa could easily be altered to address other factors at work, e.g., related to ergonomics and safety. The underlying intervention process (identifying problems, initiating preventive actions and evaluation) are coherent with basic principles of occupational health and safety practices, and may therefore also support employers in meeting legal requirements, i.e., to conduct risk assessments and encourage employees to take responsibility for their working environment.

This study also contributes with practical examples of co-designing with end-users drawing on the principles of design thinking. In this study, we used the reactions and feedback from users to adapt an adjust Susa in an iterative process in the prototyping phase. While users played a central role in the understand and prototyping phase, they were not involved directly in the explore phase. However, an important lesson was to involve users in the explore phase before developing the first prototype. Early involvement could have saved us time and money as major alternations were made to Susa in the prototyping phase, e.g., on voice and appearance.

We found that users were able to connect with Susa in a personal way, although some users were somewhat skeptical at first. Moreover, over time, users seemed to trust Susa’s instructions and expertise, and one user even felt more comfortable to speak up, because Susa is a robot and not a human consultant that may judge inputs. The anonymity and impartiality of robots have also been noted in previous research on acceptability of conversational agents that provides psychological treatment—especially among groups who are generally reluctant to seek care for mental health problems, such as veterans (Vaidyam et al., 2019).

We also found that users had strong opinions about her appearance, communication style, and personality. Overall, users favored a more human like agent with a human sounding voice and a more human like appearance rather that a more robotic type of agent. This is in line with previous research on uses perceptions of design features of embodied conversational agent in eHealth. Although results regarding gender and age differs, previous research show that users generally prefer more human like agents rather than stylized agents or cartoon like agents. However, it is still unclear if these design features will also impact behavior change (Ter Stal et al., 2020). The findings also highlight the importance of carefully considering the agents personality. We found that users disliked humor and too much chit chat but valued a more serious agent that was straight to the point. This might reflect that Susa is used in a professional setting, as previous research show that preferences depend on the specific task of the agent (Ter Stal et al., 2020).

This study also underlines several challenges and dilemmas. First, different people have different, and sometimes conflicting, preferences. Second, while using voice as a primary mean of communication has participatory and engaging affordances, users found it easier to understand and carry out instructions from Susa when given as text. Third, Susa is a simple rule-based agent that depend on prewritten commands programmed by the developer. Consequently, the user is therefore restricted to predetermined options when answering questions. In contrast, smart agents are enabled by machine learning, a type of artificial intelligence, which allows for broadening of the computer system’s capacity through its learning from data. The simplicity of Susa may prove to be its Achilles heel. Previous research show that negative feedback typically include that the users find that the agents had difficulty understanding them, that the agents were repetitive and not sufficiently interactive, and that the users had difficulty forming personal connections with the agents (Gaffney et al., 2019; Milne-Ives et al., 2020).

While several of the teams in the workplace labs successfully chose implemented interventions to strengthen their teamwork and collaborative practices, we do not know how Susa works in a real world setting. Susa advice the team to select simple problems and concrete problems to help simplify the process and increase the chance of successful implementation. However, the downside of this approach might be that the interventions are not comprehensive enough to make a measurable impact on teamwork and collaborative practices. The evaluation in stage four will help further our understanding about short-term effects on teamwork and collaboration and long-term effects on wellbeing at work. Although the developers tried not to interfere with the process, our study does not provide evidence that Susa will be useful without the presence of humans. The qualitative analyses, however, suggested that some users felt empowered to continue with solving new problems and that the experience not only strengthen their relations with their colleagues, but also boosted their self-esteem and wellbeing at work.

While this study does not allow us to draw conclusion about the effect of Susa, it does suggest that Susa could contribute to mental health *via* two distinct pathways: While the implementation of the *solutions* outlined the action’s plans could contribute to a better psychosocial working environment (and thereby better mental health), it is also possible that it is the actual *process* of coming together and implementing changes that leads to mental health and wellbeing at work. The latter hypothesis is in line with the METUX model (Motivation, Engagement, and Thriving in Users Experience) developed by Peters et al. (2018). METUX builds on *Self-determination Theory* to explain how certain design features may affect wellbeing and engagement. According to the Self-determination Theory, autonomy (e.g., feeling agency), competence (feeling able and effective) and relatedness (feeling connected to others) are essential to self-motivation and psychological wellbeing. Thus, according to this model designs should seek to support uses experience of autonomy to pursue certain goals, strengthen their sense of competence (setting them up for success) and help them stay connected with others, which is a core element in most theories of wellbeing. We are currently planning an evaluation of Susa among 30–50 workplaces, which will provide more knowledge about use in a more natural context (without the presence of researchers and consultants) and the effects on mental health, self-efficacy and collaborative practices.

## Data availability statement

The datasets presented in this article are not readily available because the data cannot be anonymized. Requests regarding the datasets should be directed to mbdn@sdu.dk.

## Ethics statement

Ethical review and approval was not required for the study on human participants in accordance with the local legislation and institutional requirements. The patients/participants provided their written informed consent to participate in this study.

## Author contributions

MN, SL, MBa, and MBu developed the project idea and designed the study. MBa and MBu developed the conversational agent and facilitated the workshops with users. SS and MBa collected the data. JA, SS, and MN analyzed the data and drafted the first version of the article. All authors contributed to the article and approved the submitted version.

## Funding

This project was funded by Velliv Foreningen grant number 18-4168.

## Conflict of interest

MBa and MBu were employed by Gnist.

The remaining authors declare that the research was conducted in the absence of any commercial or financial relationships that could be construed as a potential conflict of interest.

## Publisher’s note

All claims expressed in this article are solely those of the authors and do not necessarily represent those of their affiliated organizations, or those of the publisher, the editors and the reviewers. Any product that may be evaluated in this article, or claim that may be made by its manufacturer, is not guaranteed or endorsed by the publisher.
